# Estimating heritability using family and unrelated individuals data

**DOI:** 10.1186/1753-6561-5-S9-S34

**Published:** 2011-11-29

**Authors:** Priya B Shetty, Huaizhen Qin, Junghyun Namkung, Robert C Elston, Xiaofeng Zhu

**Affiliations:** 1 Case Western Reserve University School of Medicine, 2103 Cornell Road, Cleveland, OH 44106, USA

## Abstract

For the family data from Genetic Analysis Workshop 17, we obtained heritability estimates of quantitative traits Q1 and Q4 using the ASSOC program in the S.A.G.E. software package. ASSOC is a family-based method that estimates heritability through the estimation of variance components. The covariate-adjusted mean heritability was 0.650 for Q1 and 0.745 for Q4. For the unrelated individuals data, we estimated the heritability of Q1 as the proportion of total variance that can be accounted for by all single-nucleotide polymorphisms under an additive model. We examined a novel ordinary least-squares method, a naïve restricted maximum-likelihood method, and a calibrated restricted maximum-likelihood method. We applied the different methods to all 200 replicates for Q1. We observed that the ordinary least-squares method yielded many estimates outside the interval [0, 1]. The restricted maximum-likelihood estimates were more stable than the ordinary least-squares estimates. The naïve restricted maximum-likelihood method yielded an average estimate of 0.462 ± 0.1, and the calibrated restricted maximum-likelihood method yielded an average of 0.535 ± 0.121. Our results demonstrate discrepancies in heritability estimates using the family data and the unrelated individuals data.

## Background

The heritability of a trait is usually calculated using family data. The identified genetic variants found through genome-wide association studies account for only a small portion of heritability for most complex traits [1] compared with the heritability estimated from family data. This discrepancy in the estimates, the missing heritability, is of great interest because the sources of this difference are still unknown [1]. Recently, Yang et al. [2], using a novel statistical method, suggested that the missing heritability can be recovered using the genome-wide associations of unrelated samples [2]. Because the Genetic Analysis Workshop 17 (GAW17) data set included family data and unrelated individuals data for the same traits [3], we estimated the “heritability” of Q1 with the unrelated individuals data and estimated the “heritability” of Q1 and Q4 with the family data.

For the family data, the heritability is the narrow sense heritability, estimated with the polygenetic effect model; we conducted a George-Elston transformation [4] to estimate the heritability. For the unrelated data, the heritability is the proportion of the total variance in a phenotype that can be described by all single-nucleotide polymorphisms (SNPs) under an additive model; we estimated it using the ordinary least-squares (OLS) method suggested by Yang et al. [2], a naïve restricted maximum-likelihood (REML) method, and a calibrated REML method. In all our analyses, the heritability estimates were obtained after adjustments for age, sex, and smoking status.

## Methods

### PEDINFO and ASSOC

For the family data, we chose to use quantitative traits Q1 and Q4 of four randomly selected data set replicates (Table 1). We used the Statistical Analysis for Genetic Epidemiology (S.A.G.E.) software and the PEDINFO and ASSOC programs. The PEDINFO program calculates summary statistics about the family data set. The ASSOC program performs a family-based association test using a polygenic mixed effect model for a quantitative trait, and it estimates the heritability through the estimation of the proportion of a polygenic component to the total trait variance. In our analysis, the heritability estimates were obtained after adjustments for age, sex, and smoking status. The George-Elston transformation was applied for normality of residual distribution [4]. We did not include any genotype variables in the model.

**Table 1:** Heritability estimates for Q1 and Q4 using the family data

Replicate number	Q1		Q4	
	
	Heritability	Standard error	Heritability	Standard error
1	0.608	0.063	0.754	0.106
2	0.640	0.067	0.687	0.061
52	0.698	0.103	0.773	0.117
137	0.655	0.105	0.766	0.104

### OLS and REML estimates

For the unrelated data, we used the OLS method suggested by Yang et al. [2] and the two REML methods to estimate the heritability of Q1 with all 200 data set replicates. Here, the heritability refers to the proportion of the variance in Q1 that can be accounted for by all SNPs under an additive model [2]. We fitted the mixed effects model:(1)

where  consists of trait values of *n* unrelated individuals, , where *x_i_* = (*x_i_*_1_, …, *x_i_*_3_) consists of the sex, age, and smoking status of the *i*th individual, respectively,  consists of the effect sizes of the covariates,  summarizes genotype data of *m* unknown causal variants such that *z_i_* = (*z_i_*_1_, …, *z_im_*), and , or  if the genotype of the *i*th individual at the *j*th causal variant is *aa*, *aA*, or *AA*, respectively, *f_j_* is the frequency of allele *A* and  Here the prime indicates the transpose of a vector or matrix.

Let the effects of *m* causal variants be:(2)

where  is the variance and the residuals be:(3)

where  is the residual variance, *I_n_* is the identity matrix of order *n*,

Then the variance-covariance matrix of *y* is:(4)

where  is the genetic relationship matrix of causal SNPs and . Let *X* have the rank *r* (=*4 for the GAW17 unrelated individuals data*), and let  where  are all orthogonal eigenvectors corresponding to eigenvalue 1 of idempotent matrix . Let , , and . It follows that:(5)

where:(6)

and(7)

Note that(8)

Thus the slope and intercept of the regression of:(9)

on (*p_i_* − *p_j_*)′*G*(*p_i_* − *p_j_*) are  and , respectively. Because *G* is unknown, it is replaced with an estimate. One naïve estimate is *A*, the genetic relationship of genome-wide SNPs. Yang et al. [2] established an unbiased estimate A* for *G* by calibrating the prediction error of genetic relationship *G* of unobserved causal SNPs. Replacing *G* with A* in the regression, we can estimate the heritability as:(10)

Because this estimate is based on OLS, it does not need iteration. By replacing *G* with *A* and A* in the model given by , we can constructed the naïve and calibrated REML estimates by maximizing the likelihood of 

## Results

### Heritability estimates using the family data

In the family data, 697 individuals (202 founders and 495 nonfounders) form eight pedigrees. The pedigrees all have four generations of family members and a mean size of 87.13 individuals (range, 73–128). The pedigrees include 194 sibships with a mean size of 2.55 (range, 1–9). In the four randomly selected replicates, the heritability estimates for Q1 ranged from 0.608 to 0.698 with an average of 0.650; the heritability estimates for Q4 ranged from 0.687 to 0.773 with an average of 0.745 (Table 1).

### Heritability estimates using the unrelated individuals data

**Figure 1:**
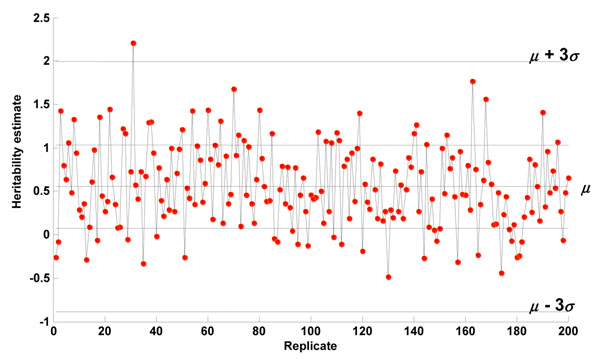
**OLS estimates of the heritability of Q1.** The estimates at many of the 200 replicates were greater than 1 or less than 0. Over the 200 estimates, the average heritability estimate for Q1 was *μ* = 0.5549 with standard error *σ* = 0.4803.

The unrelated individuals data consist of genotypes of 24,487 SNPs and 200 replicates of 697 individuals for Q1. The OLS estimates of the heritability were apparently unstable (Figure 1), because many of them were outside the interval [0, 1]. We computed the mean and standard deviation of all 200 heritability estimates, including those greater than 1 or less than 0. Over the 200 replicates, the average heritability estimate for Q1 was *μ* = 0.555 with standard deviation *σ* = 0.480 after correcting for age, sex, and smoking status.

**Figure 2:**
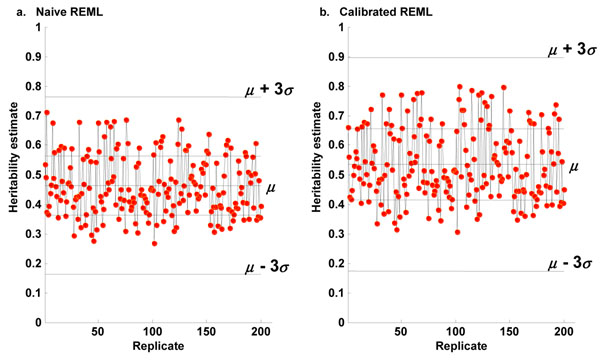
**REML estimates of heritability of Q1.** (a) The relationship *A* of genome-wide SNPs was used to estimate the relationship *G* at unobserved causal SNPs. Over the 200 replicates, the average heritability estimate was *μ* = 0.4618 with standard error *σ* = 0.0999 after correcting for age, sex, and smoking status. (b) The calibrated relationship A* was used to estimate the relationship *G* at unobserved causal SNPs. Over the 200 replicates, the average heritability estimate was *μ* = 0.5351 with standard error *σ* = 0.1206 after correcting for age, sex, and smoking status.

We found that the REML estimates for Q1 were more stable than estimates obtained using the OLS method (Figure 2). After accounting for age, sex, and smoking status, the 200 naïve REML estimates yielded an average heritability estimate of 0.462 ± 0.999, and the calibrated REML estimates yielded an average heritability estimate of 0.5351 ± 0.1206 for Q1.

We were unable to obtain REML estimates for Q4 because the convergence rate of the REML was extremely slow. We found that the convergence of the REML failed because no SNP contributed any phenotypic variation in the simulated model [3].

## Discussion and conclusions

In our analyses, we estimated heritability using both the family data for Q1 and Q4 and the unrelated individuals data for Q1. The heritability estimates for Q1 and Q4 using the family data appeared stable and reasonable. In the simulation, Q1 has a heritability of 0.575, where 0.135 is due to the 39 causal SNPs and 0.440 is due to a polygenic component, and Q4 has a heritability of 0.70 resulting from a polygenic effect. The mean heritability estimates for Q1 and Q4 with the family data were 0.650 and 0.745, respectively.

The heritability estimates using the unrelated individuals data seem less reasonable. The OLS method did not work well for the GAW17 unrelated individuals data because the method was designed for genome-wide common SNPs. In the GAW17 unrelated individuals data, most of the SNPs are rare variants and a few of them are causal variants. The genetic relationships estimated using many rare variants may be unreliable, and this results in the instability of the OLS estimates. The REML approaches appear to be more stable than the OLS method for Q1. We observed that the heritability estimates using the unrelated individuals data were less than those using the family data on average. For example, the mean of the heritability estimates for Q1 for the unrelated individuals data was 0.462 (by naïve REML), which was 0.188 less than the mean for the family data. One possible reason is that the polygenic component (0.440) in Q1 is not due to any SNPs in the GAW17 sequence data set. We should not be able to uncover the polygenic effect using unrelated samples. However, the mean naïve REML estimate (0.462) is much larger than the heritability because of the causal SNPs (0.135). The reason is that we used all 24,487 SNPs to estimate the relationships among individuals. There might be other sources contributing to the heritability estimates.

Finally, we failed to estimate the heritability for Q4 using the unrelated samples because of the convergence problem, which was the result of no genotyped exonic SNPs in the data contributing to the phenotypic variation.

## Competing interests

The authors declare that there are no competing interests.

## Authors’ contributions

PBS performed the statistical analysis of family data and HQ performed the statistical analysis of the unrelated individuals data. PBS , HQ, JN and XZ drafted and revised the manuscript. XZ conceived the project, RCE criticized and edited the manuscript. All authors read and approved the final manuscript.

## References

[B1] Manolio TA Collins FS Cox NJ Goldstein DB Hindorff LA Hunter DJ McCarthy MI Ramos EM Cardon LR Chakravarti A Finding the missing heritability of complex diseases Nature 2009 461 747 753 10.1038/nature08494 19812666 PMC2831613

[B2] Yang J Benyamin B McEvoy BP Gordon S Henders AK Nyholt DR Madden PA Heath AC Martin NG Montgomery GW Common SNPs explain a large proportion of the heritability for human height Nat Genet 2010 42 565 569 10.1038/ng.608 20562875 PMC3232052

[B3] Almasy LA Dyer TD Peralta JM Kent JW Jr Charlesworth JC Curran JE Blangero J Genetic Analysis Workshop 17 mini-exome simulation BMC Proc 2011 5 suppl 9 S2 22373155 10.1186/1753-6561-5-S9-S2 PMC3287854

[B4] George V Elston RC Generalized modulus power transformations Commun Stat Theory Meth 1988 17 2933 2952 10.1080/03610928808829781

